# A rare case of tuberculosis with motor neuron disease

**DOI:** 10.1186/1477-7819-12-381

**Published:** 2014-12-13

**Authors:** Jie Ren, Cheng Shen, Guowei Che

**Affiliations:** Department of Thoracic Surgery, West-China Hospital, Sichuan University, Chengdu, 610041 China; Department of Thoracic Surgery, Mian Yang Central Hospital, Mian Yang, 621000 China

**Keywords:** motor neuron disease (MND), tuberculosis, surgery

## Abstract

Motor neuron disease (MND) is occasionally aggravated by chronic infection. A misdiagnosed case of tuberculosis with MND is illustrated in a 45-year-old woman who underwent successful VATS wedge excision, which is presented herein. MND in an adult is a rare clinical entity. In order to facilitate the preoperative diagnosis and avoid the misdiagnosis of this disease, more etiological factors need to be considered.

## Background

Lung tumors complicated with myasthenia gravis are not commonly observed in lung cancer patients. Most of the symptoms can be relieved after resection of the tumors. Therefore, the disease with symptoms of motor and neurons as well as lung masses is clinically diagnosed as paraneoplastic syndrome. Herein, we report on a rare case of tuberculosis combined with motor neuron disease (MND) for the first time.

## Case presentation

### Case report

**Figure 1:**
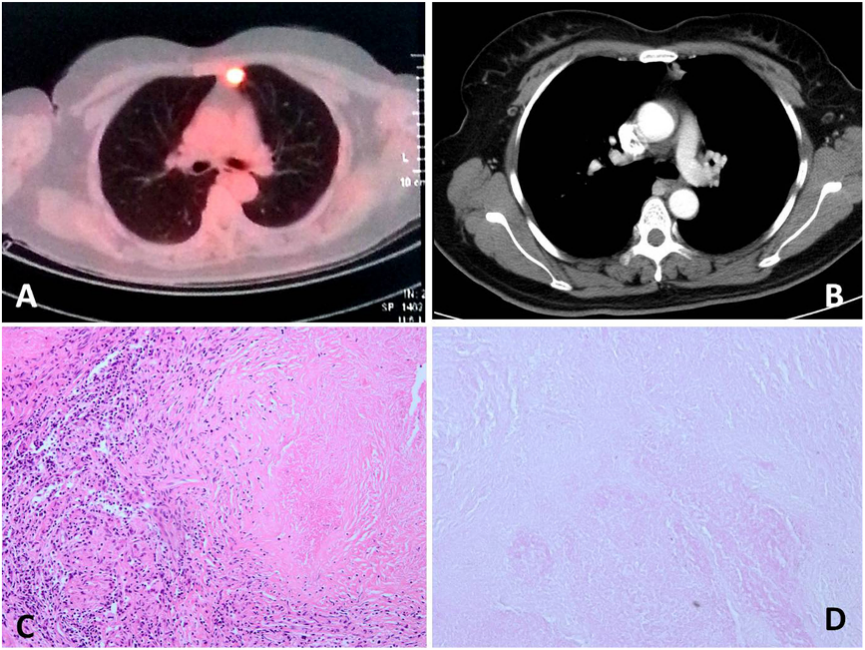
**The Positron emission tomography-computed tomography, enhanced CT and Histological features of the case. A** Positron emission tomography-computed tomography (PET-CT) showed that sugar metabolism was active in the nodules of left upper lobe. **B** The enhanced CT showed an irregular soft-tissue density nodular shadow about 1.7 × 1.4 cm in the anterior segment of left upper lobe, with several lymph nodes growing in the mediastinum. **C**, **D** The pathologic diagnosis showed granulomatous inflammation and acid-fast bacterium.

A 45-year-old woman suffered from weakness in the right upper limb without clear cause as well as an obvious reduction in distal muscle strength for more than 1 year. The symptoms extended to the left upper limb and lower limbs 6 months ago, accompanied with muscular tremors. She did not experience diplopia or sensory disorder. Dysarthrosis occurred approximately 3+ months ago, accompanied with paroxysmal dyspnea and bucking on drinking water, but without coughing, night sweat or paresthesia. She was a nonsmoker and had no exposure to any environmental fumes or dust. Physical examination revealed normal breath sounds in both of the lung fields. However, she suffered from disability of both eyeballs in looking downward, disability of the soft palate in lifting upward, dysarthrosis, atrophy in the right thenar muscles, high muscular tension in the four limbs, and tendon hyperreflexia. Proximal and distal muscle strengths were graded 5- and 4 respectively in the right upper limb, and were graded 5 and 5- respectively in the left upper limb. Muscle strengths in lower limbs were graded 5-. The bilateral pathological signs were positive. Laboratory findings were within normal limits. Skull magnetic resonance imaging (MRI) showed small ischemic focus in the left centrum semiovale. The thyroid function and the immune system were abnormal. The routine examination of cerebrospinal fluid (CSF) was normal. The glucose level was 5.79 mmol/L (normal 2.5 to 4.4) in the biochemical CSF test. CSF smears did not show acid-fast bacilli. The tuberculosis antibody test was negative. PET-CT showed the sugar metabolism was active in the nodules of left upper lobe (Figure 1A). Chest enhanced computed tomography (CT) showed an irregular soft-tissue density nodular shadow about 1.7 × 1.4 cm in the anterior segment of left upper lobe. This lobulated shadow was lightly enhanced, with several lymph nodes growing in the mediastinum (Figure 1B). Diagnosis upon admission was 1) lung cancer in the left upper lobe (?) and 2) paraneoplastic syndrome and myasthenia gravis (?). An electromyogram revealed neuronal damage in the right upper limb and the tested sternocleidomastoid muscles. A neostigmine test was negative. Diagnosis and treatment showed surgical indications on consultation in the hospital. The patient received a VATS wedge excision of the left upper lobe and lymph node biopsy. Intraoperative freezing showed that the tumors in the left upper lobe and mediastinal lymph nodes were all granulomatous inflammation with necrosis. In the pathologic diagnosis, acid-fast staining showed some positive bacilli (Figure 1C,D). Diagnosis upon discharge showed 1) tuberculoma in the left upper lobe, 2) tuberculosis in the mediastinal lymph nodes, and 3) MND. The patient recovered well postoperatively and received antituberculous treatment. The paroxysmal dyspnea and dysarthrosis were obviously improved, and the patient left hospital. The patient was followed up for 1 month without evidence of recurrence to date and the paroxysmal dyspnea and dysarthrosis were not found. All muscle strength recovered well.

## Discussion

Tuberculosis with MND is a relatively uncommon tumor in the clinic. In the present study, the patient was preoperatively misdiagnosed as lung cancer with paraneoplastic syndrome. The causes for the misdiagnosis are discussed herein. (1) The chest enhanced CT showed an irregular soft-tissue density nodular shadow about 1.7 × 1.4 cm in the anterior segment of left upper lobe. The lobulated shadow was lightly enhanced with several lymph nodes. PET-CT showed the active sugar metabolism in the nodules of left upper lobe and in the mediastinal lymph nodes. The occurrence of lung cancer with lymphatic metastasis was not excluded. The misdiagnosis was mainly caused by the overdependence on imaging, especially PET-CT, and the ignorance about the false positive of PET-CT. The rate of false positive in PET-CT is about 10%, probably due to inflammatory granulomas, such as tuberculosis, sarcoidosis, aspergillosis and histoplasmosis [1, 2]. (2) The patient had no history of tuberculosis. The symptom of this patient was mainly myasthenia, but without pulmonary symptoms, which all accorded with the manifestations of paraneoplastic syndrome of lung cancer. Lung cancer, especially small cell lung cancer, is commonly complicated with Lambert-Eaton myasthenic syndrome (LEMS) [3–5]. Since the frequently- occurring diseases are first considered during clinical diagnosis, the relevant manifestations of myasthenia in this case were misdiagnosed as the atypical manifestations of LEMS. (3) Cases of tuberculosis with myasthenia gravis were reported in the clinic [6, 7], but tuberculosis with MND has not been reported here before. Therefore, an insufficient understanding of this disease led to the misdiagnosis. MND is a degenerative disease of the nervous system without clear cause. Its pathogeny and pathogenesis are still unknown. MND is a disease of multifactorial inheritance and the potential cause is the disturbance to the RNA metabolism [8]. Besides genetic factors, other causes include excitatory toxicity of amino acids, dysfunction of glial cells, abnormal protein deposition, oxidative stress, mitochondria dysfunction, and abnormal autoimmunity. Some substances may be specifically toxic to neurons.

## Conclusions

In this case, it is unclear whether the relationship between tuberculosis and MND is occasional suppression or causality. Nevertheless, the syndromes of MND were obviously alleviated after operation. It should be further validated whether the long-term chronic inflammation of tuberculosis resulted in some substances with neuronal toxicity. The history taking and imaging diagnosis in clinic are extremely valuable for the detection and diagnosis of lung cancer and are the basis of surgical therapies, providing crucial suggestions for establishment of treatment strategies. However, with the occurrence of false positive, we should pay attention to the uncertainty of diagnostic idea and imaging, so we can accurately diagnose and reasonably treat the disease. To facilitate the preoperative diagnosis of such a rare tumor, more cases will need to be reported.

## Consent

Written informed consent was obtained from the patient for publication of this case report and any accompanying images. A copy of the written consent is available for review by the Editor-in-Chief of this journal.

## Abbreviations

CSF: cerebral spinal fluid
CT: computed tomography
MRI: magnetic resonance imaging
PET: positron emission tomography.
